# Tratamento endovascular de aneurisma de aorta abdominal com erosão de vértebra lombar associada à doença de Behçet: relato de caso

**DOI:** 10.1590/1677-5449.009416

**Published:** 2017

**Authors:** Nathalia Leslie Albanez Rodrigues de Souza, Daniel Emílio Dalledone Siqueira, Alex Aparecido Cantador, Leandro Pablos Rossetti, Giovani José Dal Poggetto Molinari, Ana Terezinha Guillaumon

**Affiliations:** 1 Universidade Estadual de Campinas – UNICAMP, Departamento de Cirurgia, Campinas, SP, Brasil.

**Keywords:** Doença de Behçet, aneurisma, coluna vertebral, lesões de coluna vertebral

## Abstract

A doença de Behçet é uma doença sistêmica, multifatorial e autoimune com diversas manifestações clínicas, entre elas o acometimento vascular. Aneurisma de aorta associado a erosão de vértebra lombar é condição rara na literatura, existindo apenas quatro relatos de caso nas bases de dados da PubMed. O presente artigo relata o caso de paciente do sexo feminino com diagnóstico de Doença de Behçet de longa data e aneurisma sacular de aorta abdominal infrarrenal com erosão de vértebra lombar. O caso foi tratado por meio de técnica endovascular com colocação de endoprótese monoilíaca e enxerto fêmoro-femoral cruzado, devido a limitações anatômicas da bifurcação aórtica. O artigo aborda a raridade desse tipo de apresentação da doença e o desfecho do tratamento e apresenta revisão da literatura sobre esse tema.

## INTRODUÇÃO

A doença de Behçet (DB) é uma doença sistêmica, multifatorial e de etiologia desconhecida, originalmente descrita pelo dermatologista turco Hulusi Behçet em 1937^1^. A síndrome foi primeiramente caracterizada pela tríade úlceras orais, úlceras genitais e uveíte. Posteriormente foram reconhecidos como parte de suas manifestações clínicas as sinovites, a vasculite cutânea, o envolvimento dos sistemas gastrointestinal e urogenital, a meningoencefalite e o envolvimento cardiovascular^2^^,^ 3. As manifestações vasculares são compostas de estenoses e oclusões, trombose ou formação de aneurismas e pseudoaneurismas, com incidência em 25% a 30% dos doentes, sendo a trombose venosa profunda de membros inferiores a mais comum^3^^,^ 4. O envolvimento arterial isolado é raro, porém está associado a complicações potencialmente fatais, principalmente secundárias à presença de aneurismas^3^^-^ 5. A aorta abdominal é o vaso mais acometido, seguido da artéria femoral e das artérias pulmonares, implicando em alto risco de complicações cirúrgicas e elevada taxa de morbimortalidade^2^^,^ 3. Os aneurismas secundários à DB respondem mal ao tratamento medicamentoso, sendo a cirurgia mandatória^5^. A modalidade de tratamento de lesões arteriais nesses doentes é, tradicionalmente, a cirurgia aberta convencional. Tal abordagem pode ser desafiadora devido a dificuldades técnicas e morbidade pós-operatória; além disso, seus resultados são afetados pela presença de atividade da doença e estão sujeitos a complicações como oclusões de enxertos e formação de pseudoaneurismas anastomóticos, sendo estes os mais frequentes e temidos.

A presença de aneurisma sacular de aorta abdominal com erosão de vértebra lombar é condição rara na DB, existindo quatro relatos de caso na PubMed até o ano de 2017^2^^,^ 6 ^-^ 8.

Para a publicação deste relato de caso, a doente assinou termo de consentimento livre e esclarecido, com dispensa de aprovação pelo Comitê de Ética em Pesquisa da Instituição.

## DESCRIÇÃO DO CASO

**Figura 1:**
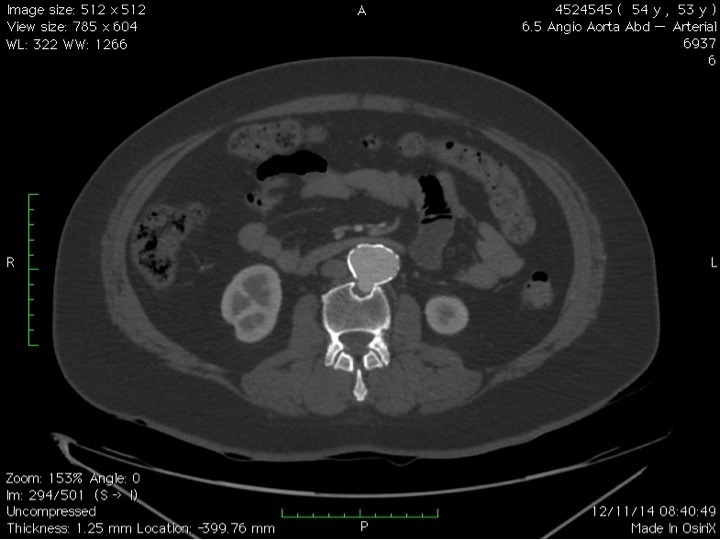
Corte axial de angiotomografia pré-operatória mostrando o aneurisma de aorta com erosão da vértebra lombar.

Doente do sexo feminino, 53 anos, com diagnóstico de DB há 20 anos estabelecido pela presença de úlceras orais e genitais e episódios recorrentes de uveíte posterior, em acompanhamento regular com a equipe de reumatologia. Apresentava ainda antecedentes de tabagismo, hipertensão arterial sistêmica, dislipidemia e doença pulmonar obstrutiva crônica. Há 1 ano iniciou quadro de dor lombar que não melhorava com analgésicos comuns ou derivados de opioides. Foi submetida a raio X simples de abdome que evidenciou imagem de calcificação em topografia de aorta abdominal e erosão óssea em topografia de terceira vértebra lombar. Foi então encaminhada ao ambulatório de Cirurgia Vascular e submetida a investigação complementar com angiotomografia de aorta para confirmação diagnóstica e planejamento terapêutico. A angiotomografia evidenciou aneurisma sacular de aorta abdominal infrarrenal com diâmetro máximo de 3,6 cm e erosão da terceira vértebra lombar (Figuras 1 e 2) e bifurcação aórtica com diâmetro de 9 mm. A paciente encontrava-se clínica e laboratorialmente em remissão da doença. Optou-se pela correção endovascular do aneurisma com endoprótese monoilíaca customizada Braile Biomédica, de dimensões 20 mm × 14 mm × 150 mm (diâmetros proximal, distal e comprimento, respectivamente) e enxerto cruzado fêmoro-femoral com prótese de dácron número 6 para revascularização do membro inferior esquerdo. Foi adotada essa estratégia devido à discrepância entre os diâmetros proximal e distal da aorta e ao reduzido diâmetro da aorta distal (9 mm), insuficiente para acomodação segura de endoprótese bifurcada. Não foi utilizado dispositivo oclusor na artéria ilíaca externa esquerda. Durante a confecção do enxerto cruzado, o aspecto das artérias femorais era habitual, não sendo observadas alterações parietais que prejudicassem a qualidade das anastomoses. Não foi observado vazamento para o saco aneurismático na angiografia de controle intraoperatória.

**Figura 2:**
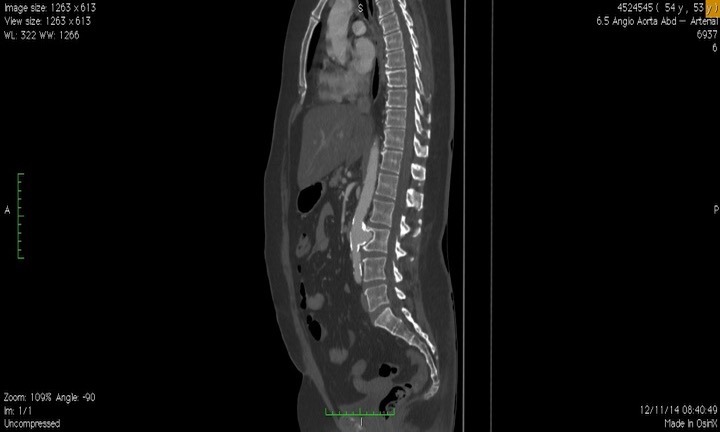
Corte sagital de angiotomografia pré-operatória mostrando o aneurisma de aorta com erosão da vértebra lombar.

**Figura 3:**
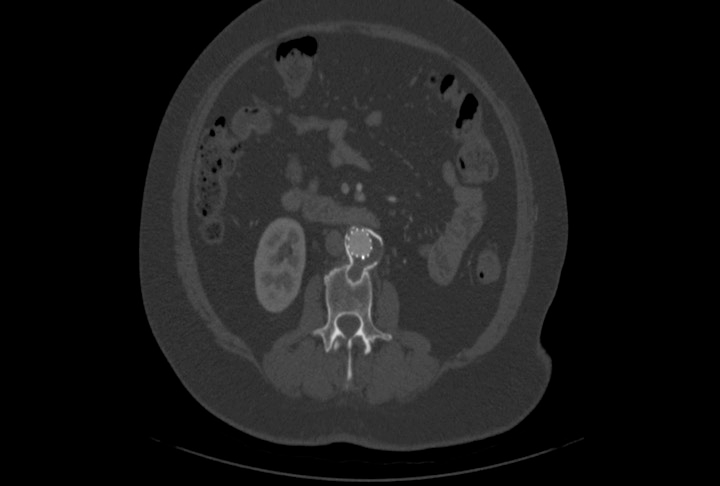
Corte axial de angiotomografia pós-operatória mostrando a endoprótese no interior do saco aneurismático, com exclusão do mesmo, sem evidência de endoleak.

Após o procedimento, a doente apresentou boa evolução clínica e recebeu alta no terceiro dia de pós-operatório. Mantém seguimento ambulatorial com evolução satisfatória e melhora completa do quadro de dor lombar. Está, ainda, em acompanhamento com a equipe de reumatologia, continuando em remissão clínica da DB, apresentando valores de velocidade de hemossedimentação de 8 mm e proteína C reativa de 0,25 mg/dL, portanto dentro da normalidade. Foi submetida a angiotomografia de controle no primeiro e no sexto mês de pós-operatório e não apresentava evidências de vazamento ou outras complicações (Figuras 3 e 4). O enxerto cruzado mantém-se pérvio e sem estenoses ou pseudoaneurismas anastomóticos (Figura 5).

**Figura 4:**
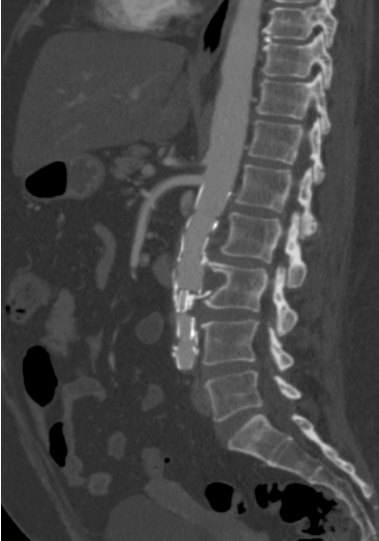
Corte sagital de angiotomografia pós-operatória mostrando a endoprótese no interior do saco aneurismático, com exclusão do mesmo e sem evidência de endoleak.

**Figura 5:**
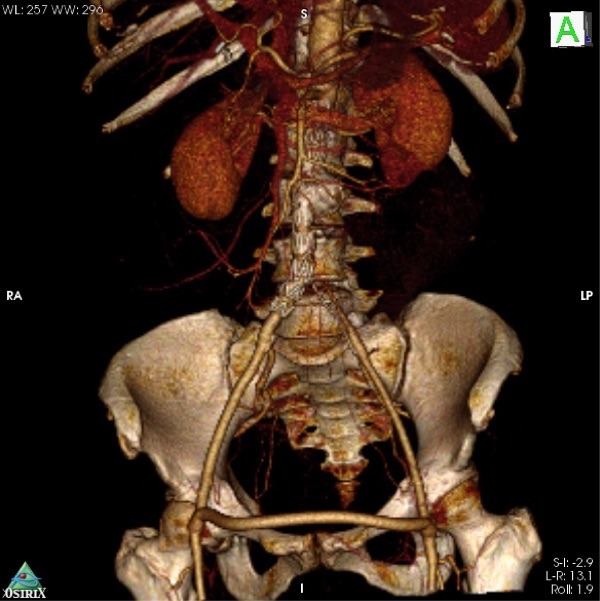
Reconstrução 3-D em software Osirix® mostrando a endoprótese monoilíaca e o enxerto fêmoro-femoral cruzado pérvio e sem evidência de pseudoaneurismas.

## DISCUSSÃO

A DB é uma doença sistêmica, inflamatória, de natureza autoimune caracterizada pela presença de lesões vasculíticas que se manifestam na maioria das vezes com úlceras orais, genitais e uveítes. Também acomete pele, articulações, vasos de qualquer calibre, tratos geniturinário e gastrointestinal, além do sistema nervoso central. Sua fisiopatologia é desconhecida, mas atualmente acredita-se que há interação de fatores genéticos – como a presença do HLA B51 – e fatores ambientais – como a infecção por bactérias do gênero Streptococcus. A DB acomete adultos jovens, com incidência entre 20 e 30 anos de idade e sem diferença entre os sexos. Embora o acometimento vascular não faça parte dos critérios diagnósticos da DB, ele está presente em 25 a 30% dos casos. A doença leva à vasculite de *vasa vasorum* e, em conjunto com a atividade inflamatória e um estado de hipercoagulabilidade, culmina em trombose e formação de aneurismas. O diagnóstico precoce da DB é essencial, devido às suas complicações potencialmente fatais. Na fase aguda da doença, deve ser investigada a presença de aneurismas arteriais, porém a formação destes pode ocorrer mesmo na fase crônica da doença. Dessa forma, deve-se atentar para sinais e sintomas que sugiram tal complicação. O acometimento da aorta na DB difere daquele nos aneurismas ateroscleróticos, pois nestes ocorre destruição e enfraquecimento acentuado da parede arterial^3^^,^ 5. As opções de tratamento cirúrgico incluem a cirurgia aberta e a endovascular. A modalidade clássica de tratamento cirúrgico nos aneurismas associados à DB é a cirurgia aberta. Tal abordagem enfrenta desafios devido a dificuldades técnicas e ao risco alto de formação de pseudoaneurismas de anastomose. Kalko et al.^5^ analisaram 16 doentes com 18 aneurismas arteriais e DB, sendo seis aórticos, cinco destes rotos. Todas as cirurgias realizadas foram enxertos com prótese de politetrafluoretileno expandido. O período de seguimento médio foi de 17 meses, e nesse intervalo dois doentes foram operados novamente devido ao surgimento de pseudoaeurismas anastomóticos e um doente apresentou novo aneurisma arterial. Dos 16 doentes, 12 estavam em remissão da doença^5^. Erentuğ et al.^1^ relataram dois casos de aneurisma de aorta roto associados à DB, operados com enxerto aortobifemoral e aorto-aórtico, com seguimento de 30 meses e sem complicações pós-operatórias. Hosaka et al.^9^ reportaram uma série de casos de 10 doentes com DB que foram submetidos a correção aberta por envolvimento arterial e observaram cinco oclusões de enxerto e cinco pseudoaneurismas durante o período de seguimento. Balcioglu et al. analisaram nove doentes com DB e aneurismas de aorta (seis infrarrenais e três suprarrenais), submetidos a tratamento cirúrgico endovascular após imunossupressão com metilprednisolona e ciclofosfamida para remissão da atividade inflamatória da DB^3^. Três doentes necessitaram de procedimento híbrido com *debranching* visceral e correção endovascular no mesmo dia. O período de seguimento foi de 40 meses, com 100% de sobrevida no primeiro ano e 88% no segundo. Não houve oclusões de endoprótese ou pseudoaneurismas, e um doente evoluiu com fístula entre o duodeno e a endoprótese, que foi corrigida com ressecção do duodeno e *patch* de omento para a endoprótese^3^. Park et al. trataram sete doentes com aneurisma de aorta com cirurgia endovascular e observaram um caso de degeneração da área de ancoramento distal da endoprótese^10^. Nitecki et al.^11^ operaram 55 doentes, sendo estes divididos em dois grupos: cirurgia aberta e endovascular. Os resultados mostraram um menor tempo de internação e menores taxas de morbidade e mortalidade no grupo tratado pelo método endovascular em comparação com o grupo submetido a cirurgia aberta.

São raros na literatura casos de aneurismas aórticos com erosão de corpo vertebral e ainda mais rara a presença de erosão vertebral secundária a aneurisma da aorta abdominal (AAA) na DB^2^^,^ 6 ^-^ 8 ^,^ 11 ^-^ 15. Lesões líticas vertebrais são associadas, geralmente, a fraturas, osteoporose, neoplasias, infecções ou estados inflamatórios. Possíveis fatores associados a dor lombar em doentes com AAA são o tamanho do aneurisma, controle inadequado da pressão arterial, dissecção aórtica e erosão de corpo vertebral^2^^,^ 16 ^,^ 17.

Os critérios diagnósticos da DB requerem a presença de úlceras orais e mais dois dos seguintes: úlceras genitais, lesões oculares típicas, lesões cutâneas típicas, ou teste positivo de patergia^18^. Acredita-se atualmente que, devido à alta taxa de recorrência da doença aneurismática nos doentes com DB, o tratamento cirúrgico aberto ou endovascular não é adequado sem terapia imunossupressora adicional^3^. O seguimento pós-operatório de aneurismas na DB deve ser regular e avaliar todas as artérias^5^^,^ 19.

Em conclusão, o envolvimento vascular na doença de Behçet aumenta sua morbimortalidade, portanto deve sempre ser lembrado e investigado nessa população de doentes. Os aneurismas aórticos com erosão de vértebra lombar são raros, porém devem ser considerados em pacientes diagnosticados com DB que apresentem dor lombar de difícil tratamento. O tratamento endovascular vem se mostrando como uma alternativa promissora à cirurgia aberta no tratamento desses doentes, porém necessita de mais estudos e tempo de seguimento para que seus resultados sejam adequadamente avaliados.
