# Supplementary material for: Tratamento endovascular de aneurisma de aorta abdominal com erosão de vértebra lombar associada à doença de Behçet: relato de caso
Source: J Vasc Bras. 2017 Apr-Jun;16(2):162–7. [Article in Portuguese] doi: 10.1590/1677-5449.009416 (PMC5915864; doi:10.1590/1677-5449.009416)
Supplement: Translation

# Translation: Endovascular repair of abdominal aortic aneurysm with lumbar vertebral erosion in Behçet’s disease: case report

**Authors:** Nathalia Leslie Albanez Rodrigues de Souza, Daniel Emílio Dalledone Siqueira, Alex Aparecido Cantador, Leandro Pablos Rossetti, Giovani José Dal Poggetto Molinari, Ana Terezinha Guillaumon

**Affiliations:** 1 Universidade Estadual de Campinas – UNICAMP, Departamento de Cirurgia, Campinas, SP, Brazil.

**Keywords:** Behçet’s Disease, aneurysm, spinal column, vertebral lesions

## Abstract

Behçet’s disease is an autoimmune, multifactorial, systemic condition with several clinical manifestations, including vascular disorders. An aortic aneurysm with vertebral erosion is rare in association with this pathology and there are only four case reports listed on the PubMed database. This article reports the case of a female patient with a long-standing diagnosis of Behçet’s Disease who developed a saccular infrarenal abdominal aortic aneurysm with lumbar vertebral erosion. Her surgical treatment consisted of endovascular repair with a monoiliac endoprosthesis and a femorofemoral crossover bypass, because of limitations imposed by the anatomy of the aortic bifurcation. This paper discusses the rarity of this presentation of the disease and treatment outcomes and offers a brief review of the relevant literature.

## INTRODUCTION

Behçet’s disease (BD) is a systemic, multifactorial disease of unknown etiology that was first described by the Turkish dermatologist Hulusi Behçet in 1937.^1^ The syndrome was initially characterized by the triad of oral ulcers, genital ulcers, and uveitis. It was subsequently recognized that clinical manifestations also include synovitis, cutaneous vasculitis, involvement of the gastrointestinal and urogenital systems, meningoencephalitis, and cardiovascular involvement.^2^^,^ 3 Vascular manifestations include stenoses and occlusions, thrombosis, or formation of aneurysms and pseudoaneurysms, with incidence rates ranging from 25 to 30% of patients, the most common of which is deep venous thrombosis of the lower limbs.^3^^,^ 4 In isolation, arterial involvement is rare, but is associated with potentially fatal complications, primarily due to development of aneurysms.^3^^-^ 5 The vessel most often involved is the abdominal aorta, followed by the femoral artery and the pulmonary arteries, so the risk of surgical complications is high and morbidity and mortality rates are elevated.^2^^,^ 3 Aneurysms secondary to BD do not respond well to drug-based treatment and surgery is mandatory.^5^ Conventional open surgery is the modality that has traditionally been used to treat arterial damage in these patients. This can be challenging because of technical difficulties and postoperative morbidity. Furthermore, outcomes are negatively impacted by the presence of disease activity and are prone to complications such as occlusion of grafts and formation of anastomotic pseudoaneurysms, which are the most common and the most devastating of these possibilities.

Formation of a saccular abdominal aortic aneurysm with erosion of lumbar vertebra is a rare condition in BD and, up to 2017, there are only four case reports indexed on PubMed.^2^^,^ 6 ^-^ 8

The patient described in this case report signed a free and informed consent form and the need for Institutional Research Ethics Committee approval was waived.

## CASE DESCRIPTION

The patient was a 35-year-old female who had been diagnosed with BD 20 years previously on the basis of oral and genital ulcers and recurrent episodes of posterior uveitis and was attending regular consultations with a rheumatology team. She also had a history of smoking, systemic arterial hypertension, dyslipidemia, and chronic obstructive pulmonary disease. One year previously she had begun to suffer from lumbar pain that did not improve with standard analgesics or opioid derivatives. A simple abdominal X-ray revealed calcification in the area of the abdominal aorta and bone erosion in the region of the third lumbar vertebra. She was referred to a Lymphedema and Angiodysplasia Clinic and supplementary investigation work up was initiated with angiotomography of the aorta for diagnostic confirmation and treatment planning. The angiotomography showed an infrarenal, saccular abdominal aortic aneurysm with a maximum diameter of 3.6 cm and erosion of the third lumbar vertebra (Figures 1 and 2) and an aortic bifurcation with 9 mm diameter. both clinical and laboratory parameters showed that the patient was in remission from BD. The treatment chosen was endovascular aneurysm repair using a customized Braile Biomédica monoiliac endoprosthesis with dimensions of 20 mm x 14 mm x 150 mm (proximal diameter, distal diameter, and length, respectively) and a femorofemoral crossover bypass with a number 6 dacron prosthesis for revascularization of the left lower limb. This strategy was adopted because of a discrepancy between the proximal and distal diameters of the aorta and the small diameter of the distal aorta (9 mm), which was too small to safely implant a bifurcated endoprosthesis. No occlusion device was used on the left external iliac artery. During construction of the crossover graft, the appearance of the femoral arteries was normal and no parietal abnormalities that would compromise the quality of the anastomoses were observed. No leakage into the aneurysm sac was observed on the intraoperative control angiography.

**Figure 1:** Preoperative axial angiotomography showing aortic aneurysm with erosion of lumbar vertebra.

**Figure 2:** Preoperative sagittal angiotomography showing aortic aneurysm with erosion of lumbar vertebra.

After the procedure, the patient exhibited good clinical recovery and was discharged on the third day after the operation. She is attending outpatients follow-up with satisfactory progress and full resolution of the lumbar pain. She is also being seen by the rheumatology team and her BD is still in clinical remission; her erythrocyte sedimentation rate is 8 mm and her C-reactive protein concentration is 0.25 mg/dL, i.e. within normal limits. Control angiotomographies were conducted 1 and 6 months after the procedure and did not show any evidence of leakage or other complications (Figures 3 and 4). The crossover graft is still patent and free from stenosis or anastomotic pseudoaneurysms (Figure 5).

**Figure 3:** Postoperative axial angiotomography showing endoprosthesis within the aneurysm sac, excluding it, with no evidence of endoleaks.

**Figure 4:** Postoperative sagittal angiotomography showing endoprosthesis within the aneurysm sac, excluding it, with no evidence of endoleaks.

**Figure 5:** 3-D reconstruction produced with Osirix® software, showing monoiliac endoprosthesis and femorofemoral crossover graft, patent and with no evidence of pseudoaneurysms.

## DISCUSSION

Behçet’s disease is a systemic inflammatory disease, autoimmune in nature, that is characterized by vasculitic lesions, in the majority of cases manifest as oral and genital ulcers and uveitis. The disease also involves the skin, joints, vessels of all calibers, genitourinary and gastrointestinal tracts, and the central nervous system. Its pathophysiology is unknown, but it is currently believed that there is an interaction between genetic factors – such as presence of HLA B51 – and environmental factors – such as infection by bacteria of the Streptococcus genus. Behçet’s disease manifests in young adults, with incidence at 20 to 30 years of age and with no difference between the sexes. Although vascular involvement is not one of the diagnostic criteria for BD, it is present in 25 to 30% of cases. The disease causes vasculitis of the vasa vasorum which, in conjunction with inflammatory activity and a hypercoagulable state, leads to thrombosis and formation of aneurysms. Early diagnosis of BD is essential because of its potentially fatal complications. The possibility of arterial aneurysms should be investigated during the acute phase of the disease, but they can also form during the chronic phase of the disease. The treating team should therefore be alert for signs and symptoms that suggest this complication. Involvement of the aorta in BD is different from atherosclerotic aneurysmal disease, because in the latter there is accentuated destruction and weakening of the artery wall.^3^^,^ 5 Options for surgical treatment include open surgery and endovascular techniques. The classical method for surgical treatment of aneurysms associated with BD is open surgery. However, this is subject to challenges related to technical difficulties and the high risk of formation of anastomotic pseudoaneurysms. Kalko et al. analyzed 16 BD patients with 18 arterial aneurysms, six aortic, five of which had ruptured.^5^ All of the surgeries were conducted using expanded polytetrafluoroethylene prosthetic grafts. The mean follow-up period was 17 months, and during this period two patients were reoperated because of development of anastomotic pseudoaneurysms and one patient developed an arterial aneurysm. Twelve of the 16 patients were in remission from the disease.^5^ Erentuğ et al.^1^ reported two cases of ruptured aortic aneurysms associated with BD that were repaired with aortobifemoral and aortoaortic bypasses and followed-up for 30 months without postoperative complications. Hosaka et al.^9^ reported a cases series of 10 patients with BD who were treated for arterial involvement with open surgery, observing five graft occlusions and five pseudoaneurysms during the follow-up period. Balcioglu et al. analyzed nine patients with BD and aortic aneurysms (six infrarenal and three suprarenal), who were treated with endovascular surgery after immunosuppression with methylprednisolone and cyclophosphamide to achieve remission of BD inflammatory activity.^3^ Three patients required a hybrid procedure with visceral debranching and endovascular repair on the same day. The follow-up period was 40 months, with 100% survival over 1 year and 88% over 2 years. There were no occlusions of endoprostheses or pseudoaneurysms, although one patient developed a fistula between the duodenum and the endoprosthesis, which was corrected by resection of the duodenum and an omentum patch to the endoprosthesis.^3^ Park et al. treated seven patients with aortic aneurysms using endovascular surgery and observed one case of degeneration of the distal endoprosthesis anchor site.^10^ Nitecki et al.^11^ operated on 55 patients, in two groups: either open or endovascular surgery. Their results showed that length of hospital stay was shorter and morbidity and mortality rates were lower in the group treated with the endovascular method compared with the open surgery group.

Cases of aortic aneurysms with vertebral body erosion are rare in the literature and even rarer when vertebral erosion is secondary to a BD-associated abdominal aortic aneurysm (AAA).^2^^,^ 6 ^-^ 8 ^,^ 11 ^-^ 15 Vertebral lytic lesions are generally associated with fractures, osteoporosis, neoplasms, infections, or inflammatory states. Possible factors associated with lumbar pain in patients with AAA are size of aneurysm, incorrect control of arterial blood pressure, aortic dissection, and erosion of a vertebral body.^2^^,^ 16 ^,^ 17

The diagnostic criteria for BD are presence of oral ulcers plus two of the following: genital ulcers, typical ocular lesions, typical skin lesions, or positive pathergy test.^18^ It is current opinion that due to the high rate of aneurysmal disease recurrence in BD patients, open or endovascular surgical treatment is insufficient without additional immunosuppressant therapy.^3^ Postoperative follow-up of aneurysms in BD should be regular and should involve assessment of all arteries.^5^^,^ 19

In conclusion, in Behçet’s disease, vascular involvement increases morbidity and mortality and should always be considered and investigated in this patient population. Aortic aneurysms with erosion of lumbar vertebrae are rare, but should be considered in patients who have been diagnosed with BD and present with difficult-to-treat lumbar pain. Endovascular treatment is proving to be a promising alternative to open surgery for treating these patients, but additional studies with longer follow-up are needed to enable adequate evaluation of the results.
